# Catalytic Hydroconversion of Model Compounds over Ni/NiO@NC Nanoparticles

**DOI:** 10.3390/molecules29040755

**Published:** 2024-02-06

**Authors:** Ting Liu, Yanxi Ma, Yakun Tang, Yue Zhang, Jingmei Liu, Xiaodong Zhou, Xiaohui Li, Lang Liu

**Affiliations:** State Key Laboratory of Chemistry and Utilization of Carbon Based Energy Resources, College of Chemistry, Xinjiang University, Urumqi 830017, China; 1635981854@163.com (Y.M.); yktang@xju.edu.cn (Y.T.); yuezhang@xju.edu.cn (Y.Z.); liujm@xju.edu.cn (J.L.); xd_zhou@126.com (X.Z.); lxhui0611@163.com (X.L.)

**Keywords:** Ni/NiO nanocomposites supported on nitrogen-doped carbon, lignite-related model compounds (LRMCs), catalytic hydroconversion (CHC), >CH–O– bond cleavage

## Abstract

The conversion of lignite into aromatic compounds by highly active catalysts is a key strategy for lignite valorization. In this study, Ni/NiO@NC nanocomposites with a high specific surface area and a vesicular structure were successfully prepared via a facile sol–gel method. The Ni/NiO@NC catalysts exhibited excellent catalytic activity for the catalytic hydroconversion (CHC) of benzyloxybenzene (as lignite-related modeling compounds) under mild conditions (120 °C, 1.5 MPa H_2_, 60 min). The possible mechanism of the catalytic reaction was investigated by analyzing the type and content of CHC reaction products at different temperatures, pressures, and times. More importantly, the magnetic catalyst could be conveniently separated by a magnet after the reaction, and it maintained high catalytic efficiency after six reuses. This study provides an efficient and recyclable catalyst for the cleavage of >CH–O bonds in lignite, thereby offering another way for improved utilization of lignite.

## 1. Introduction

Lignite is an abundant fossil resource in nature, rich in oxygenated organic matter linked by oxygenated bridge bonds, mainly in the form of >CH–O–, >C=O, and –OH [1,2,3]. Therefore, lignite must be converted into value-added chemicals through the selective cleavage of >CH–O– bonds. In recent years, catalytic hydroconversion (CHC) has received much attention due to its advantages, such as its simple process, relatively mild reaction conditions, and low environmental impact [4,5]. CHC, including hydrogenolysis and hydrocracking, has proven to be an effective way to convert lignite to small molecule compounds by cracking >CH–O bonds and to utilize aromatics further to produce clean fuels and fine chemicals [6,7,8,9].

Catalysts play an important role in the CHC of lignite and lignite-related modeling compounds (LRMCs) [10,11,12,13,14,15]. Precious metals (e.g., Ru, Rh, Pd, and Pt) have shown excellent catalytic activity in >CH–O bond hydrocracking [16,17,18,19]. For instance, Gao et al. [20] prepared a rod-shaped Au/Pd bimetallic catalyst with CeO_2_ as the support, which had high aryl ether bond cleavage activity; they achieved good results in the hydrogenolysis of dimeric model compounds. Zhang et al. [21] discovered that the synergic effect between Ru and Fe achieves high selectivity of aromatic products and catalyst stabilization in the hydrodeoxygenation of diphenyl ether over Ru_0.5_Fe_1.5_/CeO_2_ catalysts. However, the scarcity and high prices of precious metals limited their large-scale application. Therefore, research on more effective and cheaper catalysts is warranted to improve the catalytic efficiency of coal conversion. Nickel-based catalysts offer excellent catalytic performance in the hydrocracking of lignite and LRMCs due to their high activity, low cost, and low toxicity [22,23,24]. For example, Liu et al. [23] prepared mordenite (M)-supported nickel catalyst Ni/M via the precipitation method. It showed excellent activity and selectivity for the C–O bond cleavage of oxydibenzene under relatively mild conditions (160 °C, 2 MPa H_2_). With benzene as the target product, the yield exceeded 95%. Hu et al. [25] used a catalyst of nitrogen-doped carbon-loaded bimetallic Ru–Ni for the hydrocracking of various LRMCs and found that the catalyst could hydrocrack C–O bonds with high efficiency. Although the nickel catalyst has many advantages, its shortcomings, such as instability, carbon deposition, and agglomeration in the reaction process, must still be addressed [26,27]. The preparation of high-surface-area carriers and the improvement of metal dispersion on the carrier surface is a research hotspot in the preparation of high-activity hydrogenation catalysts. In recent years, carbon-based catalysts have been well-known in heterogeneous catalysis due to their advantages, such as abundant sources, low cost, excellent chemical stability, and variable structural design [28,29,30]. Meanwhile, the introduction of nitrogen atoms improves the activity and stability of the catalyst because the lone pair of electrons of the nitrogen can act as a metal coordination site to prevent the agglomeration of the active component [31,32]. Thus, nitrogen-containing carbon materials have been widely used as catalyst carriers due to their excellent chemical and electrical properties. At present, common preparation methods for Co–N–C and Ni–N–C materials include the thermal decomposition of metal-organic frameworks [33,34] and the pyrolysis of mixtures containing M, N, and C (e.g., mixtures containing melamine [35]). For instance, Song et al. [36] used the ZIF-67 one-step pyrolysis method to prepare a Co/C@N catalyst with excellent activity for the CHC of LRMCs and soluble organic matter from Naomaohu lignite. Benzyloxybenzene (BOB) was used as a LRMC under mild conditions (160 °C, 2.0 MPa H_2_). It can be completely converted into toluene and cyclohexanol after 1 h reaction. Li et al. [37] investigated the catalytic performance of nitrogen-doped carbon-loaded iron catalysts for the hydrogenolysis of BOB. The conversion of BOB was 100%, and the yields of phenol and toluene were 95% and 90%, respectively, under conditions of 240 °C, 2 MPa H_2_, and 2 h.

Although these carbon-based metal nanomaterials have excellent properties, most are difficult to produce and consume substantial energy during material preparation. Therefore, the development of a catalyst with a simple preparation method remains necessary. The Ni/NiO@NC catalyst is a composite catalyst prepared via the sol–gel method, which is characterized by a simple preparation process and a low preparation temperature. By investigating the reaction temperature, initial hydrogen pressure, and reaction time during the catalytic process of BOB, the optimal reaction conditions for the CHC of LRMCs were obtained. Furthermore, the possible mechanisms of the Ni/NiO@NC catalysts over the BOB reaction process were deduced by analyzing the types and contents of the reaction products under different conditions.

## 2. Results

### 2.1. Catalyst Characterizations

**Figure 1:**
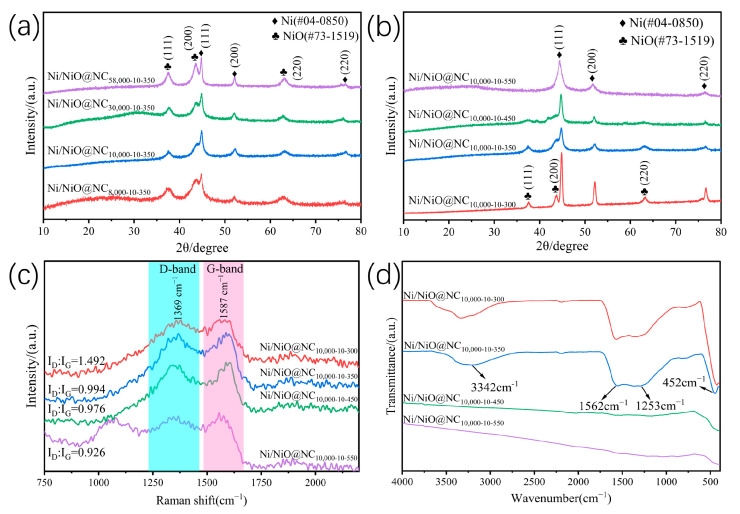
XRD patterns of different samples (**a**,**b**); Raman spectra of different samples (**c**); FTIR spectra of different samples (**d**).

XRD analysis was used to characterize the phase and crystallinity of the obtained samples. The XRD patterns of the samples with the PVP molecular weight at 8000, 10,000, 30,000, and 58,000 are shown in Figure 1a, and the XRD patterns for the samples of PVP with a molecular weight of 10,000 in the calcined temperature range of 300–550 °C are shown in Figure 1b. As shown in Figure 1a,b, Ni is present in Ni/NiO@NC nanocomposites in two forms: Ni crystalloid and NiO. The diffraction peaks at 44.5° (111), 51.8° (200), and 76.3° (220) from Ni/NiO@NC nanocomposites are attributed to the Ni crystalloid (JCPDS card no.04-0850). In addition, the well-resolved peaks at 37.5° (111), 43.5° (200), and 63.1° (220) correspond to the NiO crystalloid (JCPDS card no.73-1519). As shown in Figure 1b, the crystallinity of the NiO phase decreases with increasing temperature. Carbon components show high reducibility at high temperatures; thus, the generation of elemental Ni is due to the reduction of the carbon. Here, the NiO phase can be interpreted as the incomplete reduction of the Ni/NiO@NC nanocomposites. The nickel nanoparticle size was calculated based on XRD patterns, which are around 5 nm as shown in Figure S1. No peaks were observed from the carbon component in any of the samples because the carbon on the Ni/NiO@NC nanocomposites is amorphous, as revealed in the images of the TEM measurements. In addition, the diffraction peaks of element N were not detected by XRD due to its low content or poor crystallinity.

To check further the graphitic character of the carbon materials, the Ni/NiO@NC nanocomposites were characterized by Raman spectra. The Raman spectra for the samples in the calcined temperature range of 300–550 °C are shown in Figure 1c. All samples showed peak D (1369 cm^−1^) and peak G (1587 cm^−1^). The I_D_/I_G_ ratio shows the degree of graphitization of carbon materials, and the higher the ratio, the lower the degree of graphitization of carbon materials. The value of I_D_/I_G_ decreases from 1.492 to 0.926 with the increasing calcined temperature, indicating an increase in the degree of graphitization.

The FTIR spectra of the Ni/NiO@NC nanocomposites prepared in the calcination temperature range of 300–550 °C are shown in Figure 1d. The functional groups in Ni/NiO@NC were gradually reduced by increasing the calcination temperature. At 300 °C and 350 °C, the peak at 3342 cm^−1^ corresponds with the O–H stretching vibration, reflecting the disappearance of PVP-absorbed water molecules at high calcination temperatures (450 °C) [38]. In addition, the bands at 1562 and 1253 cm^−1^ can be ascribed to the vinyl groups of the five-membered ring and C–N stretching vibrational bands in the PVP, which are conspicuously missing in the 450 °C and 550 °C calcined materials [39,40]. A band associated with Ni–O at approximately 452 cm^−1^ provides clear evidence regarding the presence of the crystalline NiO [41,42]. As the calcination temperature increases, the infrared spectra at approximately 452 cm^−1^ become smoother due to the reduction of the NiO phase content at high temperatures, which is consistent with the XRD phase diagrams.

**Figure 2:**
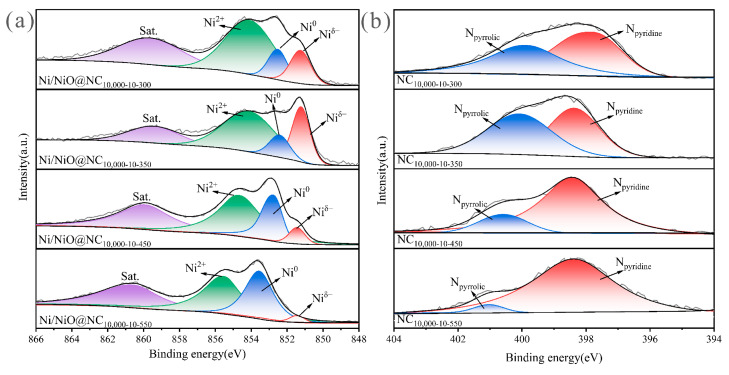
XPS spectra at (**a**) Ni 2p and (**b**) N 1s regions.

The surface elemental compositions and chemical state of elements for Ni/NiO@NC nanocomposites were deduced via XPS characterization. As shown in Figure S2, four elements, i.e., Ni, O, N, and C, were included in the Ni/NiO@NC nanocomposites. Split peaks were fitted to the N 1s and Ni 2p XPS spectra of the Ni/NiO@NC nanocomposites surface using Peak Fit software (Avantage v5.9921.0.6648), both of which were charge-corrected with the binding energy of the C–C bond (284.8 eV) in element C. Four distinct Ni species (Figure 2a) can be found at 851.3, 852.5, 854.3, and 859.7 eV in the Ni XPS spectra for Ni/NiO@NC, which can be assigned to Ni^δ−^, Ni^0^, Ni^2+^, and satellite peak [41,43,44,45,46,47]. The peaks at 852.5 and 854.3 eV of the Ni XPS spectra are attributed to Ni^0^ and Ni^2+^, proving the existence of Ni and NiO on the surface of Ni/NiO@NC nanocomposites [48,49]. In addition, the peak at 851.3 eV can be attributed to Ni^δ−^ [45,50], which may be the result of the interaction between Ni and C [51,52,53]. The δ values followed the order: Ni/NiO@NC_10,000-10-350_ > Ni/NiO@NC_10,000-10-300_ > Ni/NiO@NC_10,000-10-450_ > Ni/NiO@NC_10,000-10-550_. Two distinct peaks are observed in the N 1s XPS spectra: 400.5 and 398.4 eV, corresponding to pyrrolic-type N and pyridine-type N, respectively [50]. As shown in Figure 2b, the content of pyrrolic-type N decreases with the increase in pyrolysis temperature, and the content of pyridine-type N increases with the increase in pyrolysis temperature. The presence of different types of nitrogen species can stabilize the metal in the catalyst (similar to the role of nitrogen-containing ligands) and thereby increase the activity of the catalyst [54,55,56].

**Figure 3:**
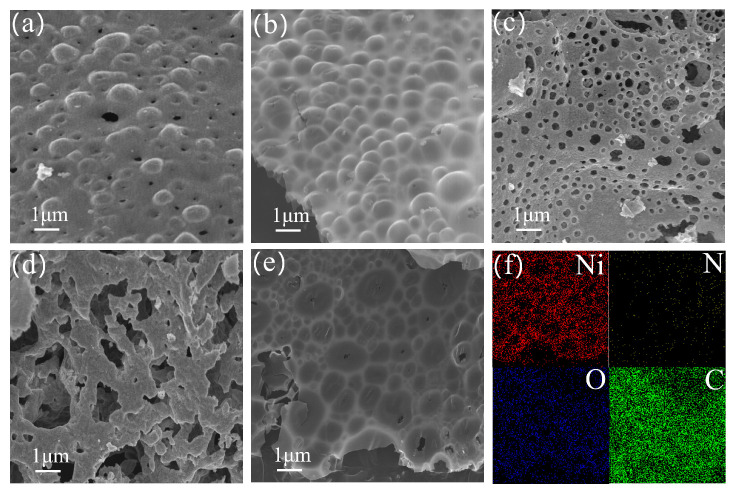
SEM images of the Ni/NiO@NC_10,000-10-300_ (**a**), Ni/NiO@NC _10,000-10-350_ (**b**), Ni/NiO@NC_10,000-10-450_ (**c**), Ni/NiO@NC_10,000-10-550_ (**d**) and SEM image and element maps of the Ni/NiO@NC_10,000-10-350_ nanocomposites (**e**,**f**).

SEM studies were conducted to reveal the surface morphologies and structural information of materials. As shown in Figure 3, with the increase in calcination temperature, the vesicle-like structure on the Ni/NiO@NC nanocomposites’ surface shows a transition from insufficient foaming to rupture of the vesicle structure. At low calcination temperatures (Figure 3a,b), the Ni/NiO@NC nanocomposites show vesicle-like structures with diameters ranging from 0.5 to 1 μm. As the calcination temperature increases, the vesicles rupture and gradually transform into a lamellar structure (Figure 3c,d), indicating that the temperature has an important effect on the growth of vesicles. In addition, Figure 3f shows the SEM images and EDS results of Ni (red), C (green), N (yellow), and O (blue). The visible highlights of different colors indicate the presence and distribution of the elements in the samples obtained, revealing that the elements Ni and O are uniformly distributed on the carbon material with a low abundance of the element N, which is consistent with the XRD results. Scanning Electron Microscope with Energy Dispersive Spectroscopy (SEM-EDS) elemental mapping was executed to observe the allotment of elements in Ni/NiO@NC nanocomposites, and the results obtained are presented in Figure S4. The SEM-EDS characterization shows that the proportions of Ni, C, O, and N are 10.03%, 59.65%, 21.88%, and 8.44%, respectively, which is in agreement with the XPS results. Meanwhile, the Inductively Coupled Plasma (ICP) results indicate that the concentration of Ni is 389 g/kg.

**Figure 4:**
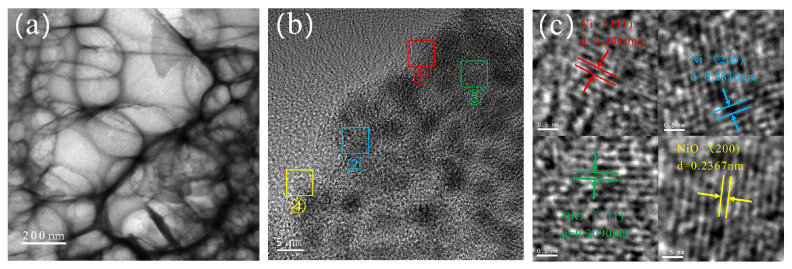
TEM image of Ni/NiO@NC_10000-10-350_ (**a**–**c**).

To observe the microstructure further, TEM analysis was conducted. The TEM image (Figure 4a) further illustrates the vesicle characteristics of Ni/NiO@NC_10,000-10-350_ nanocomposites. HRTEM was utilized to observe the Ni and NiO particles in the carbon carrier and prove the crystalline lattice in real space. Figure 4b,c displays the lattice fringes of the Ni and NiO grains. The d-spacing values of 0.241 and 0.2816 nm were indexed to the (111) and (200) crystal planes of metallic Ni, whereas those of 0.209 and 0.236 nm were assigned to the (111) and (200) crystal planes of the NiO phase.

**Figure 5:**
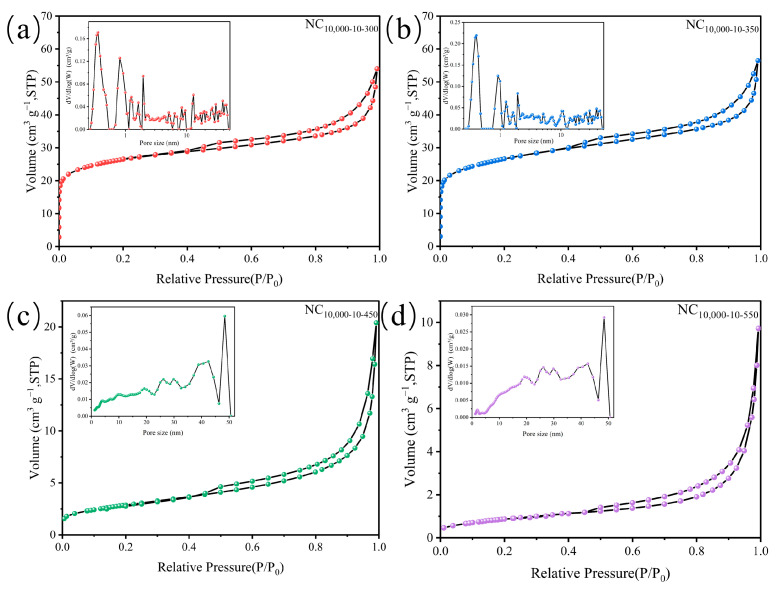
N_2_ sorption isotherm and pore size distribution of mesoporous Ni/NiO@NC_10,000-10-300_ (**a**), Ni/NiO@NC_10,000-10-350_ (**b**), Ni/NiO@NC_10,000-10-450_ (**c**) and Ni/NiO@NC_10,000-10-550_ (**d**).

As shown in Figure 5, to evaluate the surface area and porous structures of these four samples, N_2_ adsorption/desorption isotherms were acquired and analyzed on the basis of BET theory. The N_2_ isotherm shows a typical type IV curve with a sharp increase in the initial nitrogen uptake, indicating the presence of large micropores. However, with the increase in calcination temperature, the microporous structure was transformed into a mesoporous structure. The adsorption amounts of Ni/NiO@NC_10,000-10-450_ and Ni/NiO@NC_10,000-10-550_ were lower and the hysteresis lines were more evident, indicating that the catalysts had more mesopores. The Ni/NiO@NC nanocomposites’ specific surface areas were 132.7728, 133.6028, 10.3822, and 3.2702 m^2^ g^−1^. The sharp decrease in specific surface area can be attributed to the rupture of vesicles following the increase in calcination temperature, which is consistent with the SEM images.

**Figure 6:**
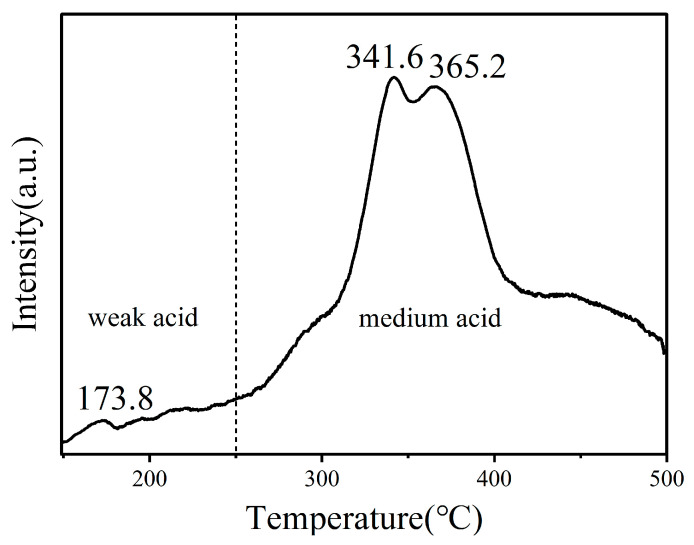
NH_3_-TPD of Ni/NiO@NC_10,000-10-350._

The NH_3_-TPD results of the Ni/NiO@NC_10,000-10-350_ nanocomposites are shown in Figure 6. The surface acid sites can be classified as weak (T < 250 °C), medium (T = 250–500 °C), and strong (T > 500 °C) based on the variation of the NH_3_ desorption peak with temperature [57]. In NH_3_-TPD of Ni/NiO@NC_10,000-10-350_ nanocomposites, three NH_3_ desorption peaks appeared at 173.8, 341.6, and 365.5 °C, confirming the existence of weak acid and moderate acid sites in Ni/NiO@NC_10,000-10-350_ nanocomposites.

### 2.2. Catalyst Screening

The application of Ni/NiO@NC catalysts to the >CH–O hydrogenolysis reaction of the BOB was investigated. As discussed in the introduction, it is essential to develop a catalyst that can be used under mild reaction conditions. As shown in Table 1, N-doped catalysts showed a significant effect on the catalytic performance. The Ni/NiO@NC_10000-10-350_ showed much higher activity than Ni/NiO@C at 120 °C (entry 8), indicating that the nitrogen species (especially electron-rich pyrrolic-type N) are favorable for promoting the hydrocracking of BOB [31]. During the preparation of catalysts, different molecular weights of PVP affect the morphology of the composite materials, which in turn affects the catalytic properties (Figure S5). Therefore, Ni/NiO@NC catalysts with different PVP molecular weights were prepared and subjected to catalytic tests. The results showed that Ni/NiO@NC_10000-10-350_ had high activity (entry 4) with 100% conversion at 120 °C. In addition, the carrier also plays an important role in the activity of the catalyst. The carrier content affects the dispersion of the active metal and thus the CHC performance of Ni/NiO@NC. A series of Ni/NiO@NC catalysts with different Ni:C ratios were prepared by modulating the carbon content and were tested for catalytic performance. At a Ni:C ratio of 1:10, the Ni/NiO@NC catalysts showed excellent performance (entry 8). Ni/NiO@NC catalysts were prepared at different calcination temperatures and investigated for the CHC of BOB. The catalytic activity of Ni/NiO@NC catalysts showed a tendency to increase and then decrease with the calcination temperature, with the best calcination temperature at 350 °C. The conversion of the Ni/NiO@NC_10000-10-550_ catalyst was 0% at 120 °C. From the SEM image (Figure 3d), it can be seen that the vesicles on the Ni/NiO@NC_10000-10-550_ catalyst appeared to be highly fragmented, which may be due to the inactivity of Ni/NiO@NC_10000-10-550_ caused by high calcination. Considering the above factors, the Ni/NiO@NC_10000-10-350_ shows the best activity among these samples, combined with the SEM, XPS, and BET characterization of the catalyst. This was due to the combined effect of the full vesicle morphology, high Ni^δ−^ content, and large specific surface area of the catalyst.

### 2.3. CHC of BOB under Different Reaction Conditions

**Figure 7:**
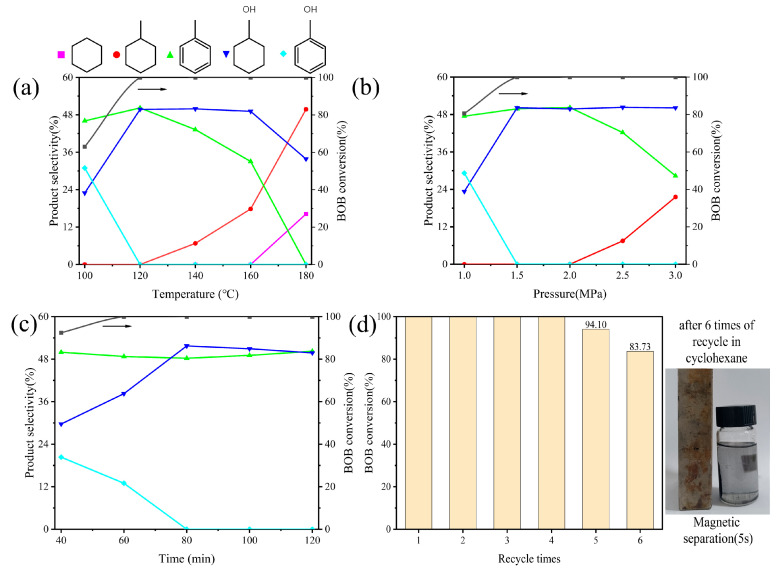
CHC of BOB under different conditions (**a**–**c**) and cyclic stability of Ni/NiO@NC_10,000-10-350_ for BOB hydrocracking (**d**).

The possible mechanism of the reaction was deduced by the catalytic hydrogenation reaction of BOB over the Ni/NiO@NC_10,000-10-350_ catalyst at different reaction temperatures, reaction pressures, and reaction times. From the catalytic hydrogenation reaction of BOB over Ni/NiO@NC_10,000-10-350_ catalyst at different reaction temperatures (Figure 7a), the cleavage of the >CH–O bond in the BOB produced one part of oxygenated compounds (phenol and cyclohexanol) and one part of nonoxygenated compounds (methylcyclohexane). When the reaction temperature was 120 °C, the selectivity of phenol in the product decreased, whereas the selectivity of cyclohexanol increased, suggesting that cyclohexanol may be obtained from the hydrogenation of phenol. When the reaction temperature was 160 °C, the selectivity of toluene in the products decreased, whereas the selectivity of methylcyclohexane increased, and the amount of change in the product was consistent. Therefore, methylcyclohexane may be obtained from the catalytic hydrogenation of toluene by the catalyst. When the reaction temperature was 180 °C, the product had 49.78% methylcyclohexane, 33.83% cyclohexanol, and 16.21% cyclohexane, suggesting that cyclohexane may be formed by the deoxygenation reaction of cyclohexanol.

To verify the above assumptions, the catalytic hydrogenation reaction of BOB by Ni/NiO@NC_10,000-10-350_ was investigated at different pressures and different times (Figure 7b,c). As shown in Figure 7b, when the reaction pressure was 1.5 MPa, the selectivity of toluene was unchanged, whereas that of phenol decreased with the increase in cyclohexanol. When the reaction pressure was increased to 3 MPa, the selectivity of cyclohexanol was essentially unchanged, whereas that of toluene decreased with the increase in methylcyclohexane. This result is consistent with the assumption. As shown in Figure 7c, the changing pattern of phenol selectivity and cyclohexanol selectivity with increasing reaction time also verified this law.

**Scheme 1:**
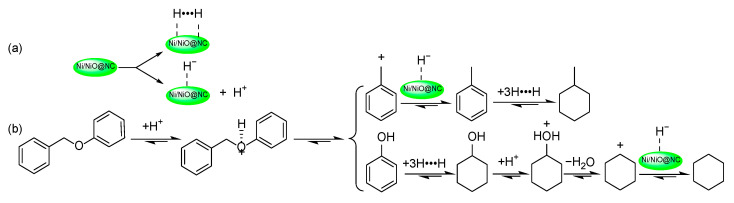
Proposed pathways for the formation of biatomic active hydrogen and H^+^ over Ni/NiO@NC (**a**) and possible pathways for the CHC of BOB over Ni/NiO@NC (**b**).

Transition metals contribute to the formation and transfer of biatomic active hydrogen (H···H) [58,59]. Ni and NiO activate H_2_ to form H···H and split H_2_ to produce mobile H^+^ and immobile H^−^, as illustrated in Scheme 1a. As shown in Scheme 1b, the >CH–O bond in the protonated BOB formed by H^+^ transfer can be cleaved directly to produce a stable phenol and a relatively stable benzyl. Then, the benzylium further abstracts the immobile H^−^ from the catalyst surface to produce toluene, followed by H···H transfer to toluene to yield methylcyclohexane eventually. Meanwhile, H···H is transferred to phenol to produce cyclohexanol. Then, H^+^ is transferred to the oxygen atom in cyclohexanol to produce protonated cyclohexanol; then, dehydration from protonated cyclohexanol and H^−^ abstraction from the Ni/NiO@NC surface by the resulting cyclohex-1-ylium followed to yield cyclohexane.

### 2.4. Cyclic Stability of Ni/NiO@NC_10,000-10-350_

The cyclic stability of catalysts is an important indicator for the performance evaluation of a catalyst. To explore the cyclic stability performance of the catalysts, a cyclic experiment of the Ni/NiO@NC_10,000-10-350_ was conducted at 120 °C, 1.5 MPa H_2_, for 60 min. As shown in Figure 7d, BOB was completely transformed after four recycles. In the two subsequent cycling experiments, the BOB conversion rate dropped to 94.10% and 83.73%, respectively. These results indicate that Ni/NiO@NC_10,000-10-350_ has good cyclic stability for BOB hydrocracking and can be rapidly recovered by an external magnet, which is a promising catalyst for application.

## 3. Materials and Methods

### 3.1. Materials

All the reagents, including Ni(NO_3_)_2_∙6H_2_O, ethylene glycol, urea, polyvinylpyrrolidone (PVP), phenyl benzyl ether, *n*-hexane, and ethanol, were purchased from Sinopharm Chemical Reagent Co., Shanghai, China.

### 3.2. Catalyst Preparation

Ni/NiO@NC catalysts were synthesized through a sol–gel method using Ni(NO_3_)_2_∙6H_2_O as a nickel source. Briefly, 6 mmol Ni(NO_3_)_2_∙6H_2_O, 18 mmol urea, and 60 mmol ethylene glycol were dissolved in deionized water (DIW, Toshima, Japan, 100 mL), and then 0.005 mmol PVP was added dropwise to the resulting green solution. The solution was stirred and heated at 110 °C until the formation of gel. Then, the collected gel was dried in a vacuum oven at 110 °C for 12 h. Subsequently, the sample was transferred to a horizontal tubular furnace for calcination at 350 °C temperature and maintained for 2 h under the protection of N_2_ atmosphere with a heat rate of 2 °C/min, which was denoted as NC_x_ (X is the molecular weight of the PVP: 8000, 10,000, 30,000, 58,000).

For comparison, a set of catalysts with nickel–carbon ratios of 1:5, 1:10, 1:15, and 1:20 were prepared by adjusting the carbon addition under the optimum Ni/NiO@NC_x_ and denoted as Ni/NiO@NC_x-y_ (y is the nickel–carbon ratio). Finally, a set of catalysts with different calcination temperatures (300 °C, 350 °C, 450 °C, 550 °C) were screened under the optimum Ni/NiO @NC_x-y_ and denoted as Ni/NiO@NC_x-y-z_ (z is the calcination temperature).

### 3.3. Catalyst Characterization

The catalysts were characterized using a Bruker Advance D8 X-ray diffractometer (XRD, Bruker, Billerica, MA, USA), Bruker VERTEX-70 Fourier transform infrared (FTIR) spectrometer (FTIRS), field-emission scanning electron microscope (SEM; Hitachi S-4800, Tokyo, Japan), transmission electron microscope (TEM; Hitachi 2600), X-ray photoelectron spectroscope (XPS; Thermo Fisher Scientific K-Alpha 1063 spectrometer, Waltham, MA, USA), microscopic confocal Raman spectrometer (Raman; Bruker Senterra Raman spectrometer), N_2_ adsorption–desorption instrument (BET; Autosorb-iQ pressure sorption analyzer, Anton Paar, Sumida, Japan), and temperature-programmed desorption of NH_3_ (NH_3_-TPD; AutoChem Model HP2950, Mack, USA).

### 3.4. Catalytic Hydrogenation and Product Analysis

BOB was used as a LRMC, and the CHC reaction was carried out in a 50 mL miniature autoclave. First, 100 mg of BOB, 20 mL of n-hexane, and 50 mg of the catalyst were added into a 50 mL miniature autoclave. After replacing air with N_2_, the miniature autoclave was pressurized with 2 MPa H_2_. Afterward, the reactor was heated to a desired temperature and held at that temperature for a period. Finally, the reaction mixture was poured out from the reactor after cooling to room temperature and separated into filtrate and residue via filtration; the filtrate was analyzed using a gas chromatograph (GC). Each experiment was repeated at least three times to ensure the repeatability of the data.

The filtrate was analyzed by a GC equipped with an SH-1701 capillary column (30 m × 250 μm × 0.25 μm), and the used catalyst was directly used for the next circulation experiment after drying.

Conversion (%) = (moles of substrate reacted/moles of substrate supplied) × 100%.

Selectivity (%) = (moles of each component/total moles of substrate reacted) × 100%

## 4. Conclusions

In this work, Ni/NiO@NC catalysts were successfully synthesized by a simple sol–gel method and employed for the CHC of LRMCs. The Ni/NiO@NC_10000-10-350_ catalyst has excellent catalytic properties for the cleavage of >CH–O bonds in BOB, achieving 100% conversion in the CHC reaction of BOB under optimum conditions (120 °C, 1.5 MPa H_2_, 60 min), due to the high specific surface area, the highly dispersed Ni/NiO nanoparticles, and the special Ni^δ−^ peak of the catalyst. The possible mechanism for the catalytic reaction process is the Ni/NiO@NC_10,000-10-350_ catalyst cleaves H_2_ to H···H and H^+^ and activation of C-O bonds resulting in complete conversion of BOB to toluene, cyclohexanol, and phenol. In addition, Ni/NiO@NC_10,000-10-350_ shows good reusability. In conclusion, this study provides the possibility of catalyzing the conversion of coal model compounds under mild conditions, which will offer a rationale for lignite utilization.

## Figures and Tables

**Table 1:** Hydrogenation of BOB over different catalysts under different conditions ^a^.

Entry	Catalysts	Conversion (%)	Selectivity (%)		
			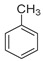	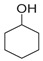	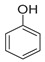
1	None	0	-	-	-
2	Ni/NiO@C_10,000-10-350_	15.74	52.37	-	47.63
3	Ni/NiO@NC_8,000-10-350_	29.25	50.53	-	49.47
4	Ni/NiO@NC_10,000-10-350_	100	47.65	52.35	-
5	Ni/NiO@NC_30,000-10-350_	68.56	42.17	23.95	33.88
6	Ni/NiO@NC_58,000-10-350_	22.4	52.4	-	47.6
7	Ni/NiO@NC_10,000-5-350_	91.26	47.52	38.51	13.97
8 ^b^	Ni/NiO@NC_10,000-10-350_	62.93	48.82	16.1	37.08
9 ^b^	Ni/NiO@NC_10,000-15-350_	24.42	51.91	-	48.09
10	Ni/NiO@NC_10,000-15-350_	100	48.9	51.1	-
11	Ni/NiO@NC_10,000-20-350_	81.54	47.26	29.53	23.21
12	Ni/NiO@NC_10,000-10-300_	43.51	47.95	-	52.05
13	Ni/NiO@NC_10,000-10-450_	37.35	49.59	-	50.41
14	Ni/NiO@NC_10,000-10-550_	0	-	-	-

Entry 2–6 were catalysts with PVP molecular weights of 8000, 10,000, 30,000, and 58,000, Entry 7–11 were catalysts with nickel-carbon ratios of 1:5, 1:10, 1:15, and 1:20, and Entry 12–14 were catalysts with calcination temperatures of 300 °C, 450 °C, and 550 °C, respectively. ^a^: Reaction conditions: BOB (100 mg), catalyst (50 mg), 120 °C, 2 MPa H_2_, IPA (20 mL), 2 h. ^b^: 100 °C, 2 MPa H_2_, IPA (20 mL), 2 h.

## Data Availability

The data are contained within the article.

## Supplementary Materials

The following supporting information can be downloaded at: https://www.mdpi.com/article/10.3390/molecules29040755/s1, Figure S1: Fitted curves at (a) Ni (b) NiO; Figure S2: XPS survey scans of Ni/NiO@NC_10,000-10-350_; Figure S3: XPS spectra at C 1s regions; Figure S4: SEM-EDS pattern of Ni/NiO@NC_10,000-10-350_; Figure S5: SEM images of the (a) Ni/NiO@NC_8000-10-350_, (b) Ni/NiO@NC_30,000-10-350_, (c) Ni/NiO@NC_58,000-10-350_.
