# Supplementary material for: Catalytic Hydroconversion of Model Compounds over Ni/NiO@NC Nanoparticles
Source: Molecules. 2024 Feb 6;29(4):755. doi: 10.3390/molecules29040755 (PMC10892770; doi:10.3390/molecules29040755)

Supplementary Materials

Catalytic hydroconversion of model compounds over Ni/NiO@NC nanoparticles

Ting Liu *, †, Yanxi Ma †, Yakun Tang, Yue Zhang, Jingmei Liu, Xiaodong Zhou, Xiaohui Li and Lang Liu *

State Key Laboratory of Chemistry and Utilization of Carbon Based Energy Resources; College of Chemistry, Xinjiang University, Urumqi, 830017, Xinjiang, PR China.

* Correspondence: liut @xju.edu.cn (T.L.); liulang @xju.edu.cn (L.L.)

† These authors contributed equally to this work.

Content

Figure S1. Fitted curves at (a) Ni (b) NiO.

Figure S2. XPS survey scans of Ni/NiO@$NC_{10,000-10-350}$.

Figure S3. XPS spectra at C 1s regions.

Figure S4. SEM-EDS pattern of Ni/NiO@$NC_{10,000-10-350}$.

Figure S5. SEM images of the (a)Ni/NiO@$NC_{8000-10-350}$, (b)Ni/NiO@$NC_{30,000-10-350}$, (c)Ni/NiO@$NC_{58,000-10-350}$.

Figure S1. Fitted curves at (a) Ni (b) NiO.

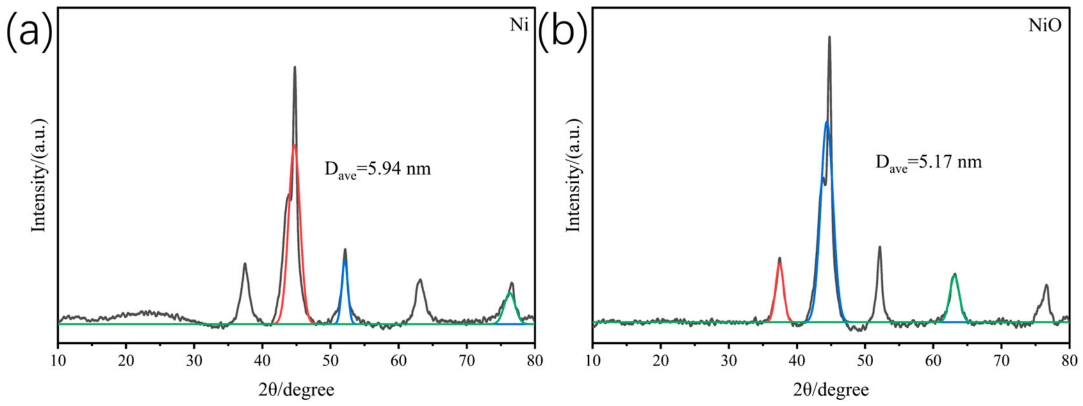

Figure S2. XPS survey scans of Ni/NiO@NC$_{10,000\text{-}10\text{-}350}$.

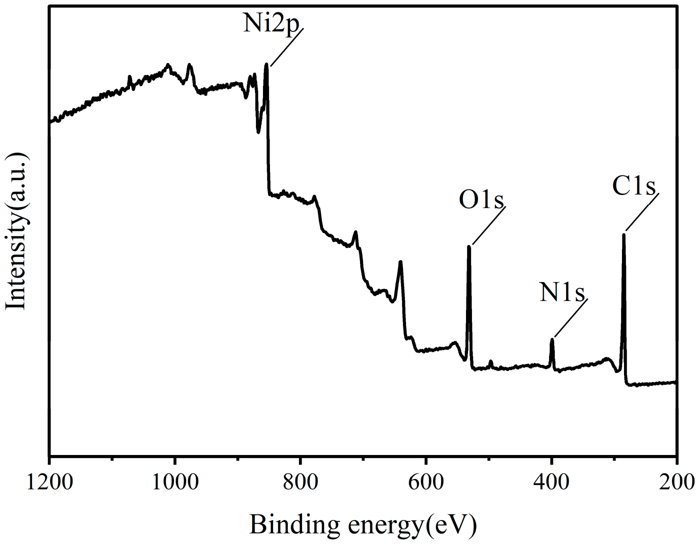

Figure S3. XPS spectra at C 1s regions.

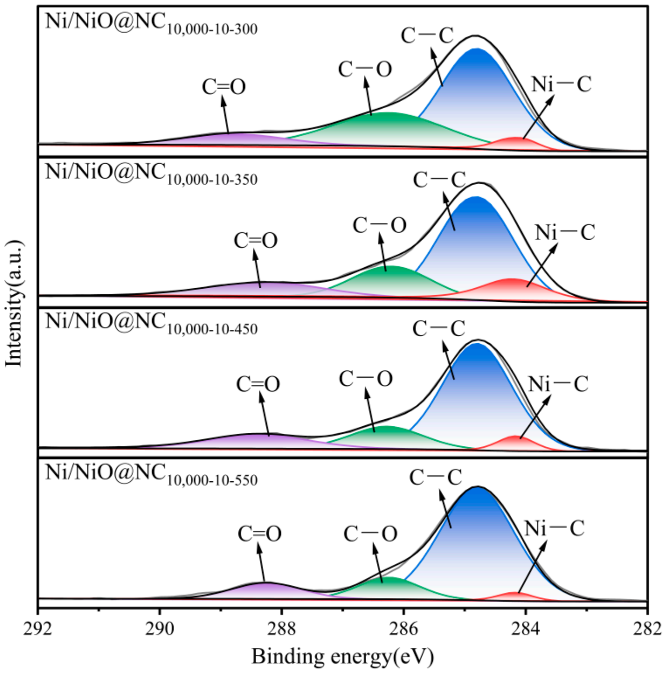

Figure S4. SEM-EDS pattern of Ni/NiO@NC$_{10,000\text{-}10\text{-}350}$.

Element	Ni	C	O	N
SEM-EDS	10.03 %	59.65 %	21.88 %	8.44 %
XPS	9.41 %	58.99 %	22.86 %	8.74 %
ICP	389 g/kg	-	-	-

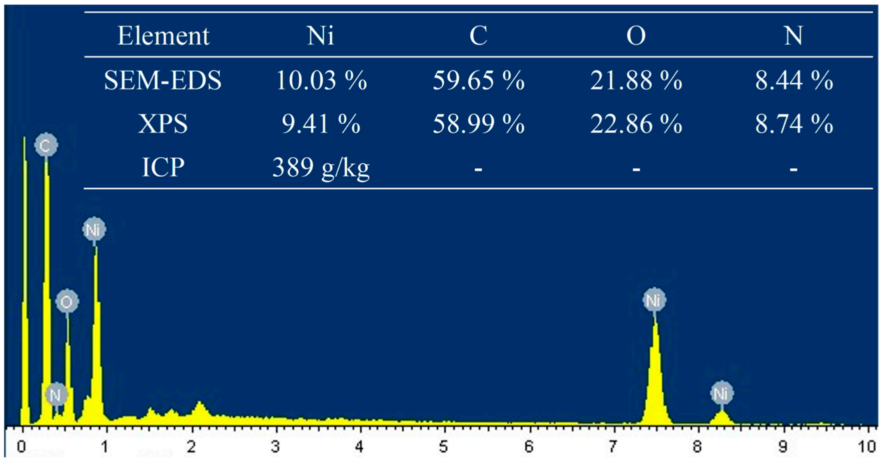

Figure S5. SEM images of the (a)Ni/NiO@NC$_{8000\text{-}10\text{-}350}$, (b)Ni/NiO@NC$_{30,000\text{-}10\text{-}350}$, (c)Ni/NiO@NC$_{58,000\text{-}10\text{-}350}$.

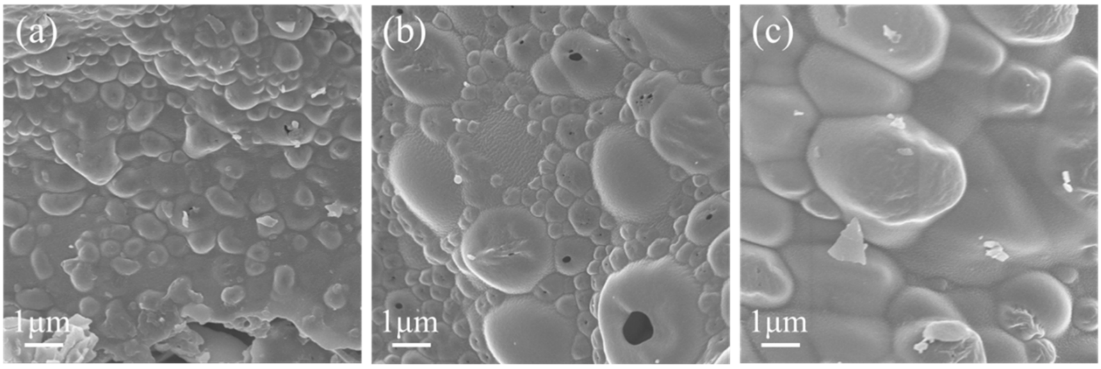

Supplement: Supplementary file 1 [file molecules-29-00755-s001.zip › molecules-2830144-supplementary.pdf]
